# Making a case for enterprise modelling as a research method

**DOI:** 10.1007/s10270-025-01343-9

**Published:** 2025-12-02

**Authors:** Sergio España, Gudrun Thorsteinsdottir, Vijanti Ramautar, Óscar Pastor

**Affiliations:** 1 Information and Computing Sciences, Utrecht University, Princetonplein 5, Utrecht, 3584 CC The Netherlands; 2 Valencian Research Institute for Artificial Intelligence, Universitat Politècnica de València, Camino de Vera, s/n, Valencia, 46022 Spain

**Keywords:** Enterprise modelling, Research methodology, Sustainability reporting, Case study, Expert assessment

## Abstract

Enterprise modelling (EM) refers to the systematic elicitation and documentation of organisational phenomena from several interrelated perspectives. Widely applied in information and computer sciences to engineer enterprises and information systems, it is rarely recognised as a research method in itself, much less outside these disciplines. This paper advocates that EM is a valid research method, within and outside its traditional application domains, highlighting its efficacy in eliciting and documenting organisational phenomena. Our study supports our claims with examples from a project investigating the application of EM as a research method to examine the intricate interplay between sustainability reporting and strategic management practices. Additionally, we have consulted experts in EM, who provided their opinions on the strengths and weaknesses of EM as a research method, and helped delineate a roadmap to foster its recognition and adoption. Throughout the paper, we emphasise the potential that EM brings to structure research and the lens that its modelling constructs provide to investigate organisational motivations, structures, processes, communications, information flow, technology, and their intricate interrelations.

## Introduction

In many scientific disciplines, we can find research projects where eliciting information about organisational phenomena and analysing it are key to answer the research questions. Sometimes, the investigation requires collecting quantitative data with the intention of revealing correlations or causal effects between certain organisational practices and organisational performance (e.g. [1]). In other occasions, the investigation is qualitative and, for instance, elicits and analyses attitudes towards some organisational aspect (e.g. [2]). At times, qualitative research projects entail unearthing structural or behavioural elements of the organisation (e.g. [3]). It is within this context that Enterprise Modelling (EM) emerges as a fitting approach, facilitating the elicitation, structuring, documentation and analysis of knowledge about the organisation, from several interrelated perspectives.

The application of EM in scientific literature is typically confined to the fields of Enterprise Engineering and Information Systems Engineering. And, within them, EM is mostly used either as the focus of the research (e.g. proposing and evaluating a set of guidelines for modelling internet of things elements in ArchiMate [4]) or as part of an information or enterprise engineering process (e.g. creating a healthcare reference model to facilitate the alignment of information and communication technology with a healthcare strategy [5]). In other words, researchers are either proposing and validating new EM methods or guidelines, or using those to engineer artefacts. EM is rarely used as a research method per se, with the purpose of answering a research question.

In this paper, we advocate for a broad recognition of EM as a useful and rigorous research method across diverse disciplines. We assert that researchers should consider EM when enterprise knowledge is the essence of their investigations and that reviewers and readers alike should embrace EM as a legitimate research method for eliciting and documenting organisational phenomena. While these claims are unlikely to come as a shock to researchers within Enterprise and Information Systems Engineering, our proposal is likely to face more resistance outside these fields. Yet, we consider EM equally valid within other disciplines, as long as the research focuses on enterprises or organisations, which is not uncommon in disciplines such as Strategic Management, Operations Management, Industrial and Organisational Psychology, Supply Chain Management, Public Administration, Corporate Social Responsibility, or Cleaner Production. As a matter of fact, this paper extends work we presented in the 16th IFIP WG 8.1 Working Conference on the Practice of Enterprise Modeling (PoEM 2023), where we presented results of a project in which we used EM to investigate the mutual influences between environmental, social and governance accounting (a.k.a. sustainability reporting) and strategic management, within large enterprises [6]. Herein, we make the following contributions:

- We present an approach to apply Enterprise Modelling methods as research methods, generalised from the approach we followed in [6], illustrating our proposal with examples from the project exploring the links between sustainability reporting and strategic management.
- We unfold and analyse the opinions of eight experts in Enterprise Modelling about (i) their impression of our proposal to consider EM as a research method, (ii) the strengths and weaknesses of EM as a research method, and (iii) what the EM community can do to promote that EM is recognised as a research method, so researchers, reviewers and readers within or outside the disciplines of Enterprise and Information Systems Engineering accept it as a valid approach.

The remainder of the paper is structured as follows: We first present an overview of the background knowledge (Sect. 2). This allows us to properly define the problem statement and the research method (Sect. 3). Then, we propose when and how an EM method could be used as a research method (Sect. 4). We present the results of the expert interviews (Sect. 5), highlighting the main opportunities and risks of applying EM as a research method, and laying out a potential roadmap that the EM community can follow to promote EM as a valid research method within and outside our discipline. We then discuss the results (Sect. 6), interpreting them in the light of earlier literature, addressing the limitations and threats to the validity, and highlight some take-aways. Finally, we conclude the paper and outline future work (Sect. 7).

While organisational managers and industry practitioners might be interested in some of our arguments or proposals, we deem the main target audience to be researchers.

## Background knowledge

### On the definition of enterprise modelling and existing methods

Sandkuhl et al. elaborate on the concept of EM and compare several definitions found in scientific literature [7, ch. 3]. Like them, we adopt the definition and conception of EM from [8]:

Enterprise Modeling (EM) is the process of creating an integrated enterprise model which captures the aspects of the enterprise required for the modeling purpose at hand. An enterprise in this context can be a private company, government department, academic institution, other kind of organization, or part thereof. An enterprise model consists of a number of related sub-models, each focusing on a particular aspect of the enterprise, e.g., processes, business rules, concepts/information, vision/goals, and actors. An enterprise model describes the current or future state of an enterprise and contains the commonly shared enterprise knowledge of the stakeholders involved in the modeling process.

Sometimes EM is also referred to as Enterprise Architecture Modelling [9]. Despite the term ’enterprise’ being used in its name, the domain of application of EM is organisations, in general, of various sizes and types, rather than being exclusive to large companies (as stated or implied in [10–12]). This encompasses a broad spectrum, including multinational corporations, small businesses, non-profit organisations, government agencies, grassroots initiatives, and informal groups. In this paper, we will indistinctly refer to enterprises or organisations, while keeping in mind this diversity.

There are plenty of EM methods and modelling languages. To name a few, Archimate [13], 4EM [7], DEMO [14], Active Knowledge Modeling [15], ARIS [16], Multi-perspective Enterprise Modeling [17]. They have in common a holistic approach to model enterprises from several interrelated perspectives (e.g. goals, processes, actors, information, business rules, technology). There are also frameworks and reference architectures, such as the Zachman Framework [18], GERAM [19], TOGAF (which includes the TOGAF Architecture Development Method, a step-by-step approach to develop and use an enterprise architecture) [20]. Some modelling methods and languages allow modelling enterprises from a more limited set of perspectives; this is the case of BPMN [21] (which focuses on the process perspective), Process Deliverable Diagrams [22] (originated in the Method Engineering community, it integrates the process and information perspectives), or iStar 2.0 (integrating the goal and actor perspectives). For the sake of brevity, we will refer to all these proposals as EM methods, even if this is not completely accurate.

### On the purposes of enterprise modelling methods

While all the EM methods enumerated above might have specific stakeholder viewpoints embedded in their guidelines and languages, all methods have similar overall purposes. Many authors have elaborated on the purposes of EM. The list is large, so we sample a few (find more details in the companion technical report [23]): re-engineering the enterprise [10, 11, 24], communicating among stakeholders [11, 12], achieving proper integration (e.g. information system interoperability) within the enterprise and with external actors [24, 25], as a reasoning tool for evaluating the application of new technology to the enterprise [10], developing information systems that are aligned with the enterprise [11, 12, 24]. Some purposes are related to investigation and research; for instance:

- Diagnosing a disorder or malfunction of any type (regarding material, information, control, decision or any other flows) [24].
- Better understanding of how the enterprise should work and how it really works [24].
- Acquiring knowledge about the enterprise from different stakeholders [11].
- Creating, documenting and maintaining a complete and multifaceted view of an organisation or business [11].
- Analysing an enterprise [25]

Yet, this possibility of using EM as a research method has, so far, mostly been used to investigate organisations within engineering projects, e.g. when redesigning an organisation to better adapt it to a changing environment, or when developing information systems to support the organisational goals and processes. In Sect. 2.3, we clarify some notions related to research, and in Sect. 2.4, we explore the rare cases where EM has been used as a method to answer scientific research questions.

### On design science and its two kinds of research problems

According to Hevner et al. [26], Design Science is a scientific research paradigm in which research projects are often triggered by a problem-solving process, during which some artefact needs to be created while, at the same time, acquiring knowledge and understanding of the design problem or its solution. Wieringa [27] also highlights the dual nature of Design Science and distinguishes two types of problem-solving cycles that correspond to two kinds of research problems, namely (i) the design cycle, that corresponds to a design problem, and (ii) the empirical cycle, that corresponds to knowledge questions. The design cycle concerns the design of artefacts intended to help stakeholders. It is therefore meant to tackle **design problems** that call for a change in the real world and requires analysing actual or hypothetical stakeholder goals (e.g. engineering a modelling tool for Entity-Relationship Diagrams, that is founded on a rigorous metamodel, to be used in educational settings [28]). A specific design is one possible solution among many, and a treatment proposed as a solution should be evaluated by its utility with respect to the stakeholder goals. The empirical cycle aims at producing answers to **knowledge questions** about the world; for instance, about the artefact in context (e.g. what modelling constructs of Entity-Relationship Diagrams have widespread usage and should be part of the modelling language abstract syntax). The answer to a knowledge question is a proposition that can be evaluated by truth, and it is subject to fallibilism. Both design problems and knowledge questions are often expressed as research questions.

### On the use of enterprise modelling methods for scientific research purposes

We found Kirikova’s work [29] on the representational and explanatory capabilities of EM enlightening. Although her paper does not explicitly characterise EM as a research method, it does provide a theoretical examination of the capabilities of enterprise models, drawing from Aristotle’s explanatory principles. We have found some examples of papers outside Information and Computer Sciences that have applied (fragments of) EM methods as a research method, with or without explicit recognition of this fact. For instance, Shukla et al. [30] propose the use of Role Activity Diagrams [31] to elicit and document detailed healthcare pathways and use the resulting models as input for identifying problematic phenomena, modelling decision-making processes, and analysing and simulating pathway variations. In [32], the authors propose a method to facilitate converting tacit knowledge into explicit knowledge. They draw inspiration from existing EM methods to propose a visual conceptual modelling language for knowledge acquisition, where the resulting models can be queried, navigated, exported and transformed into transformed into Concept Network models [33]. Sawitria et al. [34] have used Business Model Canvas^1^ [35] to investigate product diversification strategies in Indonesian enterprises of the food sector. Gerber et al. [3] adopt Zachman Framework for Enterprise Architecture (ZFEA) as an explanatory information systems theory. They apply ZFEA in seven case studies with South African enterprises, to structure the interview protocol. They recorded and transcribed the interviews and later coded them in Atlas.TI using ZFEA elements as a base reference as well as relevant themes that emerged from the interviews. In this way, each transcript was mapped to the different elements of the ZFEA, facilitating the identification of possible patterns of focus during enterprise growth. They also aggregated results to produce a heatmap and identify how knowledge about the holistic organisation and its underlying parts is relevant for growth. Herein, we will refer as Enterprise Modelling research method (EMRM) to an EM method used as a research method.

## Setting the stage: a definition of the problem and our approach

### Problem statement

**Context: Enterprise Modelling as a method of research, rather than a subject**. First, we would like to remind the reader of the distinction between EM being the subject or the method of research:

- *Conducting research on the domain of EM*. The majority of the focus of the EM scientific community is placed here. In this context, EM researchers typically (a) propose new EM theories [36], methods [7] and tools [37] (typically a *design problem*), or (b) gather knowledge to understand the context of EM education or practice, or to evaluate the performance of such methods and tools (typically a *knowledge question*), e.g. using action research [38], surveys [39], experiments [38], usability evaluations [40], or case studies [41].
- *Using EM as a research method (EMRM)*. As discussed in Sect. 2.4, there are not many examples of papers where a research question inquiring certain knowledge about organisations has led the authors to apply EM as part of their research method. In such cases, we could expect researchers to apply EM to elicit phenomena about organisations and later analyse that information to answer the research question. This paper focuses on this situation.

**Stakeholders: scientific researchers within or outside Information and Computer Sciences**. The main stakeholders are scientific researchers who need to investigate phenomena related to organisations. As stated above, organisational phenomena is a common domain of analysis in many disciplines. While some of these disciplines might fall within the same scientific field as EM, for instance, Business Informatics (e.g. [42]), many more fall outside, such as Strategic Management (e.g. [43]), Operations Management (e.g. [44]), Industrial and Organisational Psychology (e.g. [45]), Supply Chain Management (e.g. [46]), Public Administration (e.g. [47]), Corporate Social Responsibility (e.g. [48]), Cleaner Production (e.g. [49]), Social Work (e.g. [50]), Medicine (e.g. [51]), Education (e.g. [52]).

Here we would like to distinguish disciplines within the field of Information and Computer Sciences from disciplines in fields that are more distant from Enterprise Modelling.

- *Scientific researchers within Information and Computer Sciences*. In this multidisciplinary field, researchers typically (i) develop hardware, algorithms, and software, (ii) formulate and test concepts and theories about the former, taking also the actors and context of their application into account, and (iii) engineer methods and tools to conduct research or consultancy in those domains. Some researchers in this scientific field belong to the EM community or are knowledgeable of EM methods. And it is even more frequent that information scientists and computer scientists are versed in at least some of the perspectives that are regarded as part of Enterprise Modelling, for instance, any of the flavours of Information Modelling (e.g. Database Design [53], Conceptual Modelling [54], or Ontology Engineering [55]) or Process Modelling (ranging from Flowcharts intended to represent software structure [56], to business processes intended to represent organisational behaviour [57]). Therefore, such researchers are likely aware of the usefulness and strengths of these languages. Furthermore, many researchers might have used information modelling as part of the conceptualisation of a data analysis project or the formulation of a theory. The idea that EM can be used as a research method is more likely to fall on fertile ground in an audience of information and computer scientists and might even feel like preaching to the choir when presented to the EM community (an expectation later confirmed during the expert assessment interviews). Authors, reviewers and readers in this field are likely to be receptive and easily regard EM as a valid and rigorous research method. The situation might be less favourable outside this field.
- *Scientific researchers within other scientific fields*. Nowadays, stakeholders in fields outside Information and Computer Sciences have some knowledge of and experience with information and communication technology, if only because they are users of hardware and software in their professions or personal lives. But this does not imply that the research methods in Information and Computer Sciences are widely accepted or recognised as producing relevant results with rigour. Moreover, it is unlikely that researchers in other scientific fields know about EM. It is logical to consider that researchers in such fields will not resort to EM as a research method because they have not been trained on EM, not even in projects where EM might be suitable to answer their research questions. It is also our expectation that reviewers and readers from other scientific fields will even frown upon when reviewing or reading a paper that claims to have used EM as a research method. For several reasons: (i) they will have never heard of it, (ii) it is not conventional in their journals, and (iii) even if they are explained how EM is applied and what it produces, they might regard it as useful within consultancy projects but not rigorous or validated enough to deserve being considered as a research method. In sum, we expect more resistance to our proposal in fields outside Information and Computer Sciences.

To account for these differences, in this paper we will sometimes explicitly contextualise our argumentations.

**Goals: eliciting, documenting and analysing phenomena about organisations, in order to answer a knowledge question**. As argued above, in many research projects, researchers investigate organisations. Their methods vary depending on the discipline. Even when they opt for qualitative research methods, they often resort to interviews, surveys, document inspection, or case studies mixing some of the former elicitation methods. Later, the researchers often perform thematic analysis or grounded theory. Some researchers within Information and Computer Sciences know of the capabilities of EM to guide the elicitation and documentation of organisational phenomena. The majority of times EM is applied in a research project, it is the subject of research (e.g. [58]. When it is applied as a research method, it is most of the times employed to engineer organisations or information systems, rather than to answer a knowledge question, a research question.

**Problematic phenomena: Researchers are missing the opportunity to apply EM as a research method**. Any lack of structure and explicitness when documenting organisational data complicates the validation of the data to ascertain that it is accurate and faithfully represents the phenomena that a research project is set to investigate. It also complicates its analysis and interpretation. EM offers ways to structure the data collection protocols with a holistic view of the constituents of an enterprise, and it offers modelling languages that allow documenting the elicited data unambiguously and with a level of detail that adapts to the needs of the investigation. The problem is that, by failing to resort to EM, researchers might experience a lack of guidance in eliciting and documenting data from enterprises, leading to sub-optimal results in terms of research process rigour, and results completeness and validity.

### Research method

**The research questions** addressed by this paper are the following:

- **RQ1: How can an Enterprise Modelling (EM) method be used as a scientific research method?** We generalise our earlier experience applying EM as a research method, illustrating the proposal with a running example.
- **RQ2: What are the strengths and weaknesses of applying an Enterprise Modelling method as a scientific research method?** We explore the benefits and drawbacks of EM when applied to answer a knowledge question, as well as the success and failure factors of those endeavours.
- **RQ3: What can the Enterprise Modelling community do to promote Enterprise Modelling as a valid research method within and outside our discipline?** We wish to see EM recognised as a valid and rigorous research method by members within and outside our community. We assume that many members of the EM community share this wish (the EM experts we have interviewed confirm this point). We explore the challenges ahead of us and opportunities to achieved the deserved recognition.

**Fig. 1:**
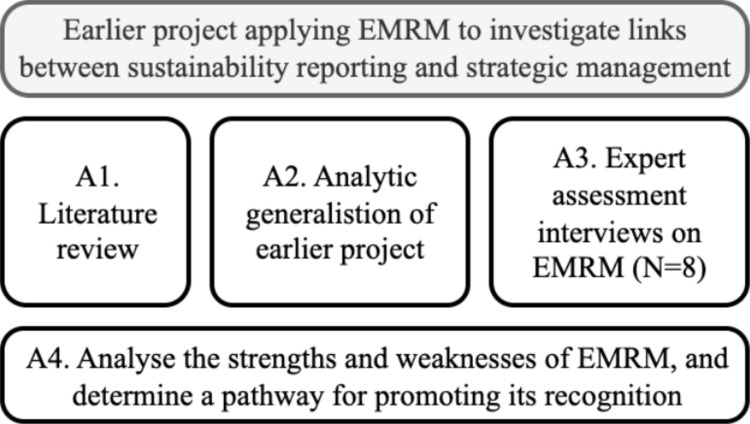
Overview of the research method as a set of research activities

Figure 1 presents the research method applied to answer the research questions.

To initiate exploring our idea that EM can be used as a research method (EMRM) and to the establish the background for this, we have resorted to scientific literature (activity A1 in Fig. 1). We have performed a literature review (i) to collect the declared purposes of EM and identify which of them are related to research (we report on this in Sect. 2.2), and (ii) to search for the few research papers that have applied EMRM (see Sect. 2.4). While we have not applied a systematic protocol, the results serve the purpose of providing background knowledge.

In [6], we adopted an hands-on approach, by applying EMRM in one of our own research projects. We had committed ourselves to investigating the links between sustainability reporting and strategic management, and we considered the use of EM as a useful approach for detecting the existence of such links in enterprises. This is represented at the top of Fig. 1 as a greyed-out activity. While the prior study served a specific substantive purpose, here our objective is to extract and formalise case-independent guidance on when and how EM can be used as a research method. This constitutes an analytic generalisation [59] of the earlier project (the results of activity A2 are reported in Sect. 4). We illustrate our proposal with examples drawn from the earlier project.

**Fig. 2:**
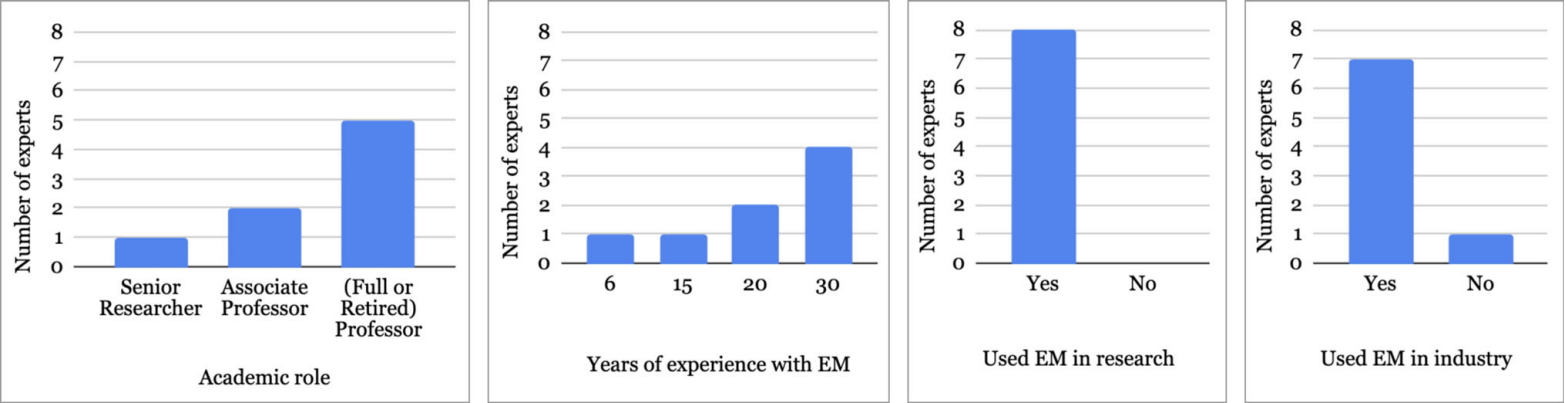
Demographics of the participants of the expert assessment interviews

Encouraged by the results of the earlier project, we also became interested in discussing our proposal with representatives from the EM community. We have conducted expert assessment interviews ( A3 ) with eight experts in EM. We selected the interviewees through convenience sampling, among Programme Committee members of PoEM 2023. All experts are (or have been) academics; see their roles in Fig. 2. Their experience with EM range from 6 to 30 years.^2^ They have all applied EM in research projects and they have all published scientific papers in this discipline. All but one have applied EM in industrial contexts, either as part of their research projects (when they collaborated with industrial partners) or by offering companies their professional consulting services. We conducted semi-structured interviews in which (i) we asked for their informed consent to participate in this research, (ii) we elicited the demographic information, (iii) we briefly explained our claim that EM can be used as a research method and asked them to react to this proposal, (iv) we asked for their opinion about the strengths and benefits that EM has as a research method, (v) about its weaknesses, limitations or failure factors, and finally (vi) we offered the expert to provide any final remarks. The interviews took place in person or via teleconference. They were recorded, with permission of the interviewees.

Finally, we conducted a qualitative analysis to examine the strengths and weaknesses of using EM as a research method and to identify actions the EM community could undertake to promote its recognition as a valid research method. Interview recordings were transcribed using the online service Transkriptor, followed by a thorough manual verification to correct errors, ensure accurate attribution, and anonymise identifying information. The verified transcripts were analysed using thematic analysis [61] in a two-cycle coding procedure [62]: (1) deductive structural coding with predefined codes aligned to the interview questions and (2) inductive pattern coding in which codes emerged from the participants’ statements, revealing salient themes and cross-cutting patterns. The results of this analysis are presented in Sect. 5.

## Applying Enterprise modelling as a research method

Enterprise Modelling (EM) enables researchers to represent complex organisational realities through formalised, expressive models that capture the entities, relationships, processes, and rules within an enterprise. It offers a way to explore and explain how different organisational elements interact and contribute to broader phenomena. However, using EM as a rigorous qualitative research method means using it not only to create models of organisational artefacts, but also as a structured approach to collect, document, analyse, and interpret data relevant to organisational phenomena.

Unlike more traditional qualitative methods that often rely on thematic coding or narrative analysis, EM provides a systematic framework rooted in explicit ontologies (i.e. formal specifications of the key concepts and their interrelations). This ontological commitment could help clarify terminology, reduce ambiguity, and support the consistency and coherence of the analysis.

Using EM as a research method aligns with qualitative inquiry goals, such as understanding complex social and organisational contexts in depth and capturing the richness of real-world phenomena. At the same time, EM’s structured and formalised nature could help address common challenges in qualitative research, including the need for transparency, replicability, and clear communication of findings.

In the subsections that follow, we discuss when and how EM can be effectively applied as a research method. We illustrate these points with a running example based on a research project on sustainability reporting and strategic management that was published in PoEM 2023 [6] and that we introduce next.
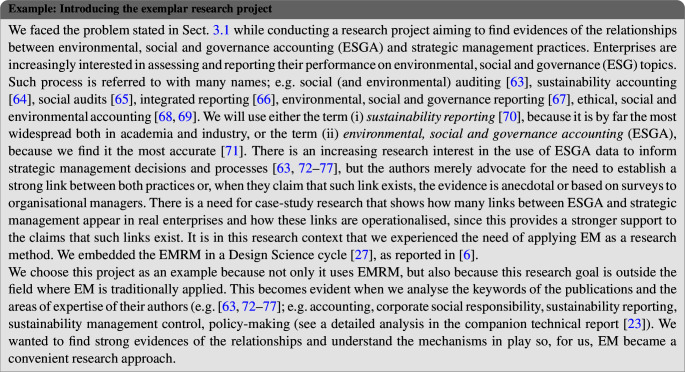


### When to use enterprise modelling as a research method

Enterprise Modelling (EM) is particularly well-suited for research that involves exploring complex organisational structures, processes, and interactions where formalised representation can reveal underlying patterns and mechanisms. Researchers should consider using EM as a research method when their research questions require one or more of the following actions:

- **Capturing and analysing organisational complexity.** When the focus is on understanding how multiple organisational elements (such as roles, departments, processes, resources, and information flows) interrelate, EM helps to create comprehensive models that make these connections explicit.
- **Clarifying domain ontologies and terminology.** EM supports the development of precise, shared vocabularies through metamodels that define concepts and their relationships. This is valuable in domains where ambiguity or inconsistent terminology hampers understanding and communication; for instance, in research project situations where researchers (and perhaps also practitioners) from multiple disciplines collaborate.
- **Structuring qualitative data in a formal framework.** When qualitative data from interviews, documents, or observations need to be organised systematically, EM provides a scaffold to translate narrative information into well-defined constructs and relations. Optionally, researchers can integrate EM with qualitative analysis, using constructs from the EM metamodel as codes in tools such as NVivo, ATLAS.ti, MAXQDA, Dedoose, QDA Miner, HyperRESEARCH, Transana, RQDA, Taguette, and CATMA.
- **Investigating processes and mechanisms behind phenomena.** EM is effective for revealing how processes operate and how various factors influence each other within an enterprise, enabling the generation of explanatory models rather than just descriptive accounts.
- **Supporting stakeholder engagement and validation.** Visual and formal models produced through EM facilitate collaboration with practitioners, enabling them to better understand, critique, and validate the research findings.
- **Bridging qualitative and quantitative research.** Occurrences of EM metamodel construct instantiations can be counted, if this contributes in any way to answering the research questions. But, more importantly, while EM is primarily qualitative, its formal structure can support the formulation of hypotheses and the identification of variables, potentially guiding future quantitative inquiry.

This list is not intended to be exhaustive; it reflects understandings informed by our research and the broader EM community’s experience. With a growing adoption of EM as a research method, these criteria are expected to evolve. Some generic research questions for which EM might be appropriate as a research method are (i) “How are the enterprise structures in this type of organisation?” (ii) “What is the prevalence of this practice within this domain?” (iii) “What are differences between organisations with regard to this aspect?” Nonetheless, EM may not be universally appropriate for all qualitative research contexts. Specifically, research focusing primarily on subjective experiences, emotional states, or cultural phenomena that lack clear organisational structures or formal representations may be less amenable to EM. Furthermore, effective application of EM requires a certain level of expertise in modelling techniques and a commitment of effort that may not be justified in highly exploratory or small-scale studies with narrow scopes. These considerations and the practical strengths and limitations of EM as a research method have been examined in detail through expert assessment interviews, as discussed in Sect. 5. In summary, EMRM is most advantageous when the research goal is to develop clear, structured, and ontologically grounded models of organisational phenomena, particularly when understanding relationships, interactions, and underlying mechanisms is essential.
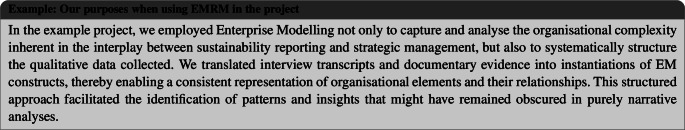


### How to apply enterprise modelling in research

Applying Enterprise Modelling (EM) as a research method involves a systematic process that integrates modelling activities with established practices for data collection, validation, and analysis. The following steps outline a typical approach to incorporating EM into a research design:

1. Understand the domain. Start by investigating the context of the research, and collecting the background knowledge. This might range from conducting a literature review to substantiate the research questions, to pilot investigations to find arguments or evidences that invite a deeper analysis. This investigation is also useful to later define requirements and constructs for the EMRM.
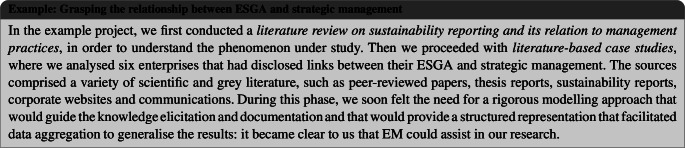


2. Define the Ontological Commitment. Clearly stating the modelling constructs used to represent organisational phenomena constitutes what is known as the ontological commitment [78]. This refers to the implicit or explicit assumptions made by a modelling language regarding the existence and nature of entities within a domain. Clarifying these assumptions enhances transparency and interpretability of the resulting models, and explicitly communicates how data will be structured as well as what types of relationships or concepts will be represented [79].

Determining the ontological commitment within a research project of our scope can be approached in several complementary ways:

- Reviewing existing conceptualisations or metamodels relevant to the domain of study, to identify the constructs they provide and the assumptions embedded within them. These artefacts serve as useful starting points, but they do not always exist or might not be readily available.
- Conducting systematic literature reviews or qualitative analyses to extract relevant domain constructs, either informally or through rigorous coding processes.
- Iteratively developing and testing preliminary or candidate ontological commitments, using them to capture or interpret empirical data from case studies or interviews. This allows for the adjustment of the ontological scope based on the suitability of the constructs to represent observed phenomena adequately. The trade-off here lies in the required effort.
- Engaging domain experts to validate or refine conceptual boundaries further enhances the accuracy and relevance of the commitment, though this may be more feasible in larger projects

Ultimately, defining the ontological commitment is not a one-off task but an evolving process that may require revisiting as the research unfolds, especially when new insights or complexities arise. This iterative refinement aligns with established qualitative methods (e.g. grounded theory).

In any case, documenting the ontological commitment explicitly supports reproducibility and critical evaluation. It also facilitates communication between interdisciplinary teams by providing a clear conceptual basis. Moreover, later on, the ontological commitment will inform the selection and application of the EMRM.
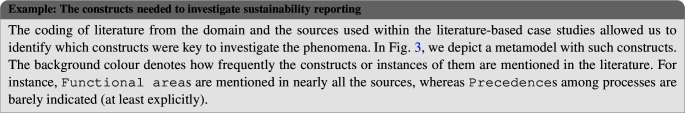

**Fig. 3:**
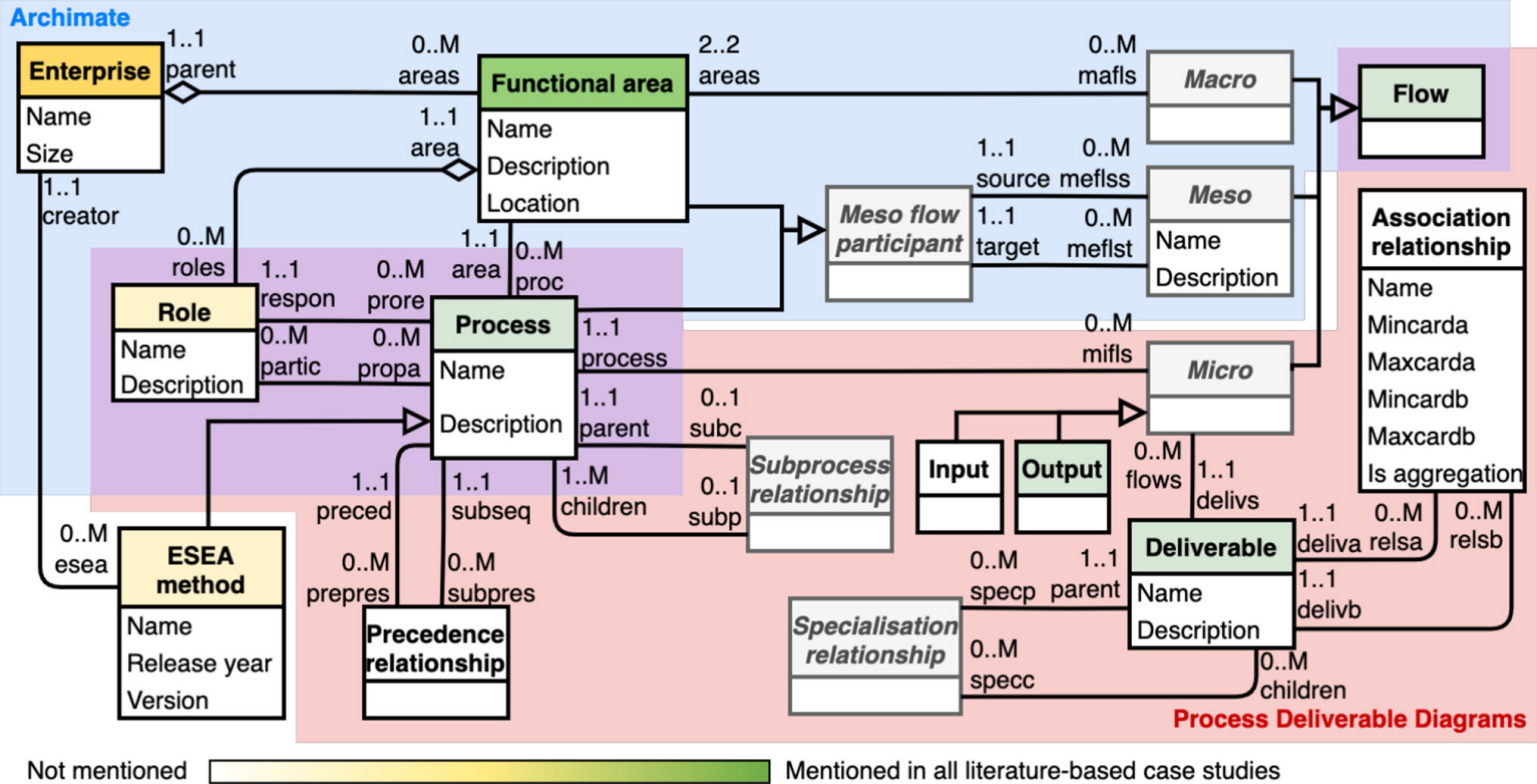
Metamodel containing the constructs resulting from the qualitative analysis of the sources. The background colour of the metaclass header denotes the frequency with which the construct appears. The greyish classes with their name in italics were added afterwards, to better structure the model

3. Select an Appropriate EM Method. To inform the decision of which EM method to choose, it is recommended to identify the requirements for such method, as well as the constructs that are relevant to the research. The objective is choosing an EM method whose underlying ontological commitment (i.e. metamodel) aligns with the ontological commitment of the research project (discusses above). Consider the method’s ability to represent the organisational elements and relationships relevant to the research questions. Selection criteria may include the following:

- Expressiveness of the modelling language. Its underlying ontology (e.g. metamodel) should be suitable for the intended investigation.
- Comprehensibility by stakeholders. A modelling language with a clear semantics and an easy-to-grasp notation can help participants engage in discussions, as well as interpret and validate the models.
- Compatibility with the research team’s expertise. Prior experience with a given EM method can tip the balance towards that method.

Of course, other decision-making criteria can be considered; Bock et al. provide a comparative analysis of several EM methods [80] and Petersen et al. present criteria for selecting an EM method [81]. Researchers may then adopt an existing EM method or compose a custom method by combining method fragments. In the latter case, such combination can either be performed through an ad hoc approach (e.g. [82]), through ontological alignment (e.g. [83]), or through method integration [84] or engineering [22, 85] approaches (e.g. [86]). When combining methods, the researchers should ensure that the resulting modelling language maintains coherence and quality [87, 88].
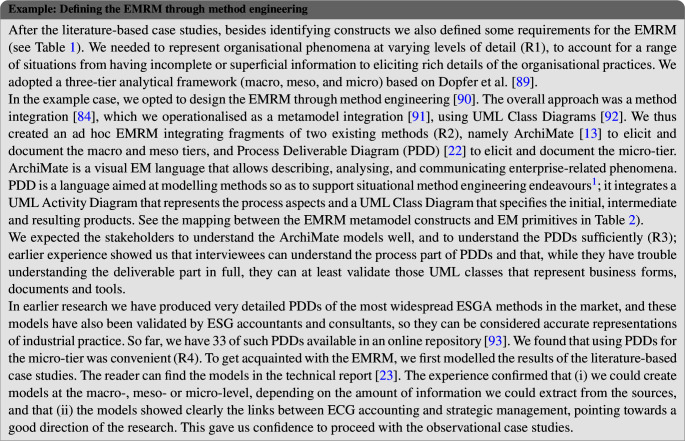
3

**Table 1:** Requirements for the enterprise modelling research method, defined after the problem investigation

Id	Requirement
R1	The EMRM should allow us to define the links between ESGA and managerial practices at different levels of detail, allowing for different depths of analysis, depending on the amount of information that the case studies provide
R2	The models resulting from applying the EMRM should be understandable by stakeholders, to a degree that allows them to provide feedback
R3	The EMRM can be made of fragments of existing EM methods
R4	The resulting EMRM or its constituent fragments, should be compatible with our competencies and earlier research we have conducted in the field of ESGA

**Table 2:** Mapping of EMRM metamodel constructs to EM language primitives. This mapping also determines the two shapes in the background of Fig. 3 where blue represents the ArchiMate EM method fragment, red refers to Process Deliverable Diagrams, and purple represents both

Metamodel construct	ArchiMate	Process Deliverable Diagram
Enterprise	Business actor	
Functional area	Business actor	
Role	Business actor	Role
Process	Business process	Activity
Flow	Flow	Process-deliverable arrow
Precedence		Activity edge
Deliverable		Class
Relationship		Association relationship
Specialisation		Specialisation relationship

4. Elicit Data Using Qualitative Methods. Gather empirical data using techniques such as interviews [94], document analysis [95], group model building [96], participatory modelling workshops [97], or observations [98]. These techniques provide the substantive input for model construction and interpretation [99, 100]. EM does not replace these qualitative methods but rather structures and enhances their outcomes by (i) guiding the elicitation process (e.g. informing the design of interview protocols), and (ii) classifying elicited data according to the agreed ontological commitment. Group model building and participatory modelling workshops also enable stakeholder engagement and validation.

It is also important to consider the availability and confidentiality of data sources. Organisational stakeholders may require researchers to sign non-disclosure agreements restricting the publication of sensitive information (such as the name or location of the enterprise). Such constraints are generally manageable, provided sufficient evidence remains available to support the research findings.
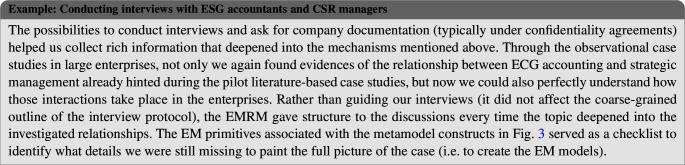


5. Construct Enterprise Models. Translate the elicited data into enterprise models by applying the selected EM method. Enterprise Modelling allows researchers to formalise organisational processes, identify relationships, and integrate multiple perspectives of the organisation into coherent representations. For the enterprise modelling process and specific modelling guidelines, the researchers should refer to the handbooks of the chosen EM method. Of course, the enterprise models can be created in parallel to the data elicitation (e.g. during a participatory modelling session) or sequentially.

The models should remain closely aligned with the research questions, avoiding unnecessary detail or scope creep. Similar to efforts to avoid over-engineering in enterprise models [101], maintaining the focus when using EM as a research method helps ensure that modelling efforts provide meaningful insights relevant to the research questions, rather than expending resources on irrelevant or overly granular aspects. Previous accounts of enterprise modelling projects highlight the risk of falling into these pitfalls and help avoid some mistakes (e.g. [102]).
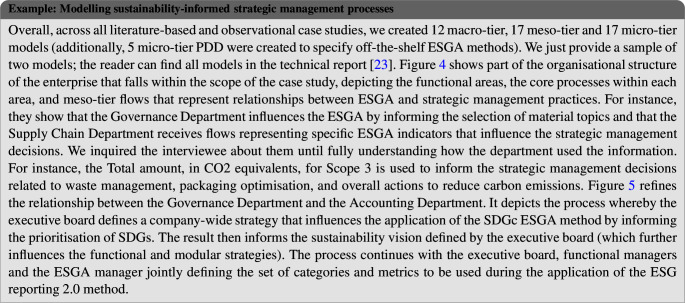

**Fig. 4:**
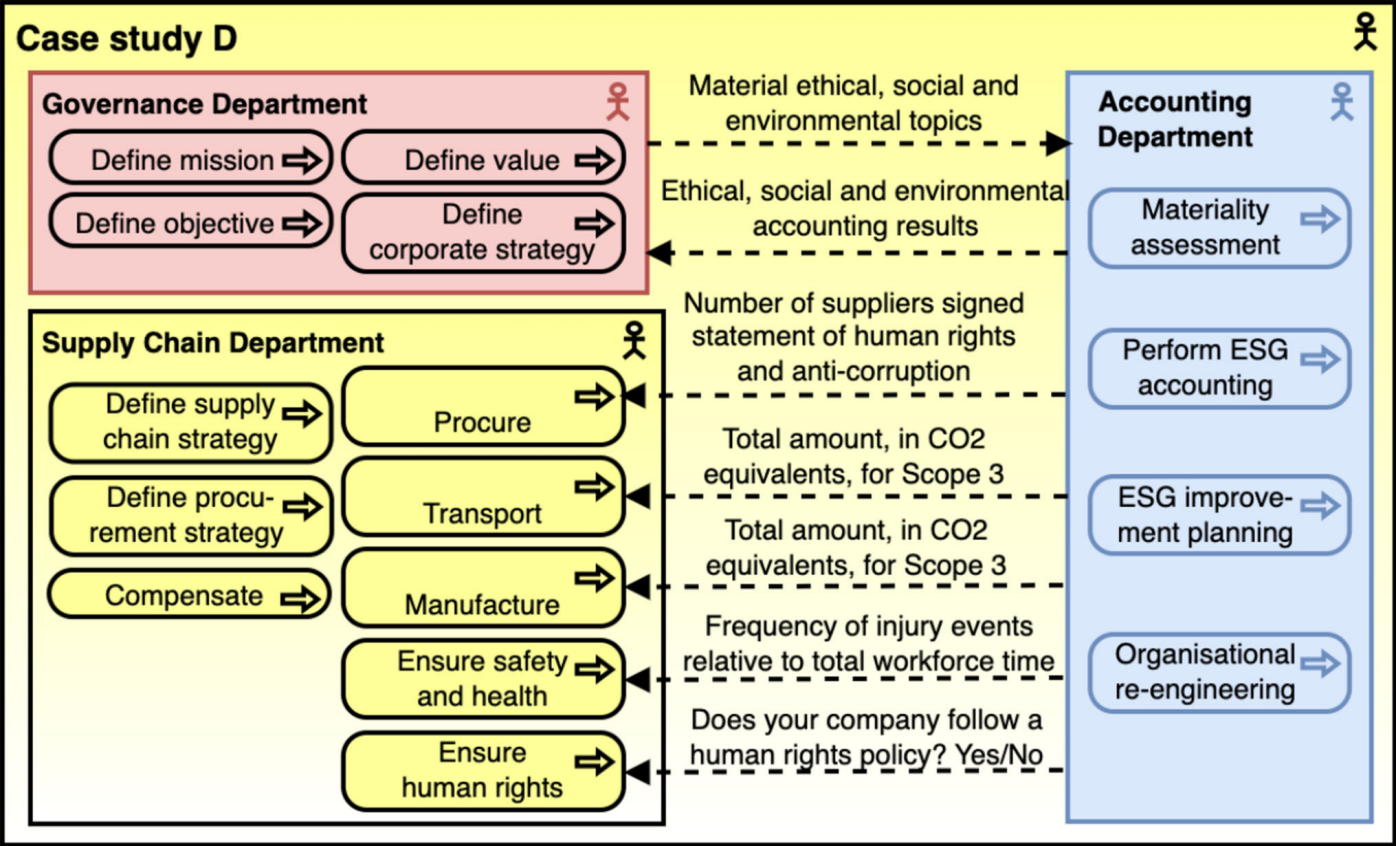
ArchiMate model depicting the meso-tier information flows from and to the Accounting Department that relate ESGA practices to managerial practices, with the focus put on the Supply Chain Department

**Fig. 5:**
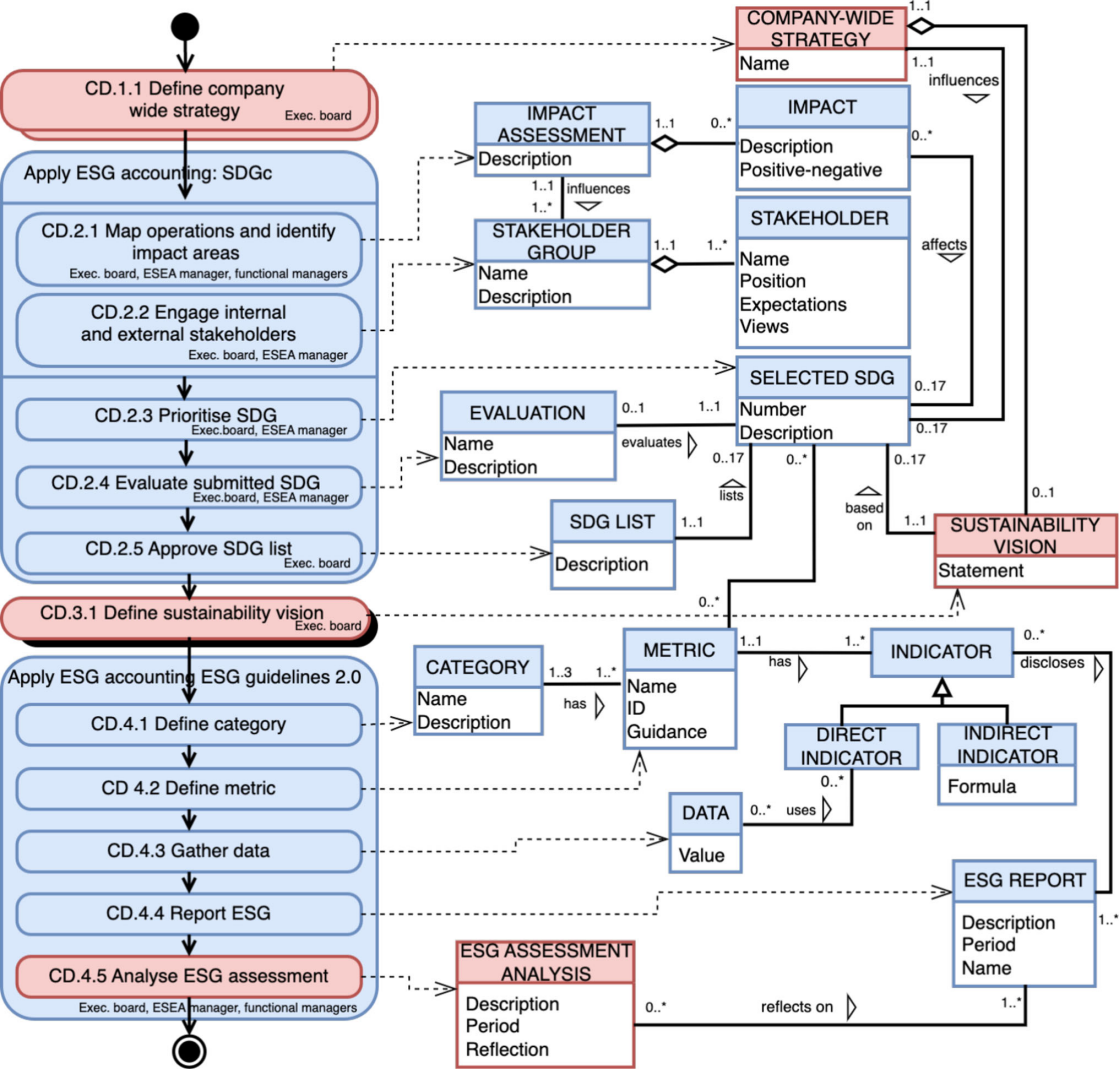
Process Deliverable Diagram depicting a company-wide process from case study D whereby the strategic managers influence the ESGA practices. Elements with blue background are primarily the responsibility of the ESG Accounting department, elements in red background are primarily related to the Governance management

**Fig. 6:**
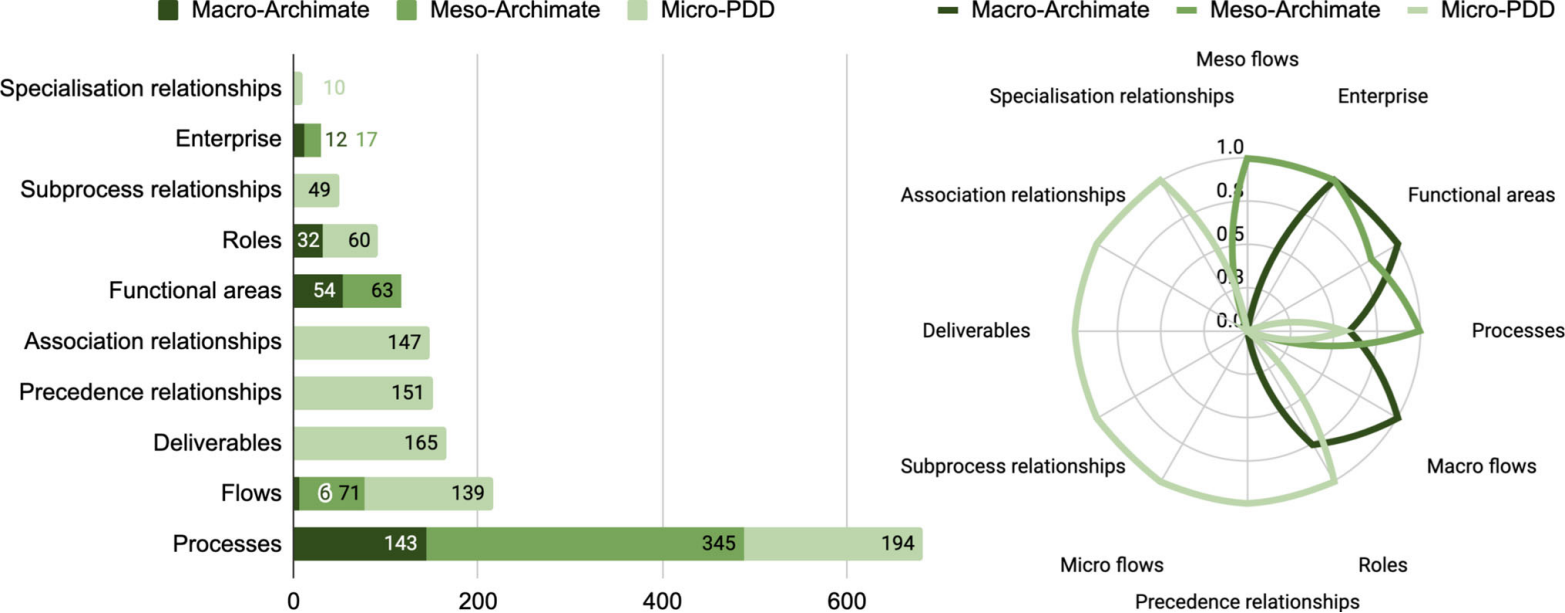
Overview of the use of EMRM metamodel constructs across the models of the three tiers created during this research. Left: stacked barchart showing the total number of instances of each construct considering all models. Right: radarchart representing how each tier focuses on a subset of the constructs; scores are calculated using normalised averages of the number of instances of each construct per model

5. Triangulate Findings and Validate Enterprise Models. Triangulating findings and validating models enhances the internal validity of research results [103]. Where possible, we recommend triangulating the findings by comparing data from different sources (e.g. interviews supported by policy documents or internal reports) [104]. Engaging with participants or stakeholders to validate the constructed models can be performed through feedback sessions or workshops, which help confirm accuracy and foster mutual understanding between researchers and practitioners [105]. However, these activities require additional participant time, which may not always be feasible. Researchers should therefore consider participatory modelling approaches, which, although also time-consuming, incorporate ongoing (implicit or explicit) validation throughout the modelling process [97].
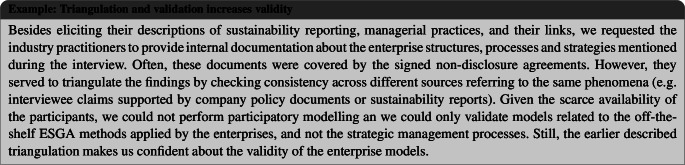


6. Analyse and Interpret the Enterprise Models. The completed models serve as the foundation for analysis. Researchers can use them to identify patterns, contradictions, bottlenecks, or opportunities, depending on the research aims. Because enterprise models formally capture organisational phenomena, they facilitate rigorous comparisons across cases, support “what-if” scenario explorations, and provide explanatory power regarding systemic effects and emergent behaviours. The formalisation inherent in enterprise modelling also enhances transparency and replicability of the analysis.

Moreover, when enterprise modelling is combined with other research methods (whether qualitative, e.g. surveys, interviews, quantitative, e.g. statistical or correlation analysis, or mixed-method approaches, e.g. Q methodology), the resulting integrated findings can offer a more holistic and nuanced interpretation of the studied phenomena [106]. This triangulation strengthens the validity of conclusions and can reveal insights not accessible through any single method alone. Anyhow, the strengths and challenges of combining several research methods are well known to the Information and Computer Sciences [107].

Crucially, the analysis and interpretation of the enterprise models allow researchers to directly address the formulated research questions by grounding their answers in a structured and systemic representation of the organisation and its context. This linkage should ensure that conclusions are tightly coupled with the empirical data represented in the models, thereby strengthening the validity and relevance of the findings.

The interpretive process benefits greatly from iterative reflection and involvement of domain experts, who help contextualise findings and ensure that interpretations align with practical realities, thereby increasing the utility and impact of the research.

Finally, it is important that the researchers clearly state the extent to which the results are generalisable. For this purpose, for instance, they can refer to the types of generalisability defined by Lee and Baskerville [108], or the generalisability levels defined by Yin [109].
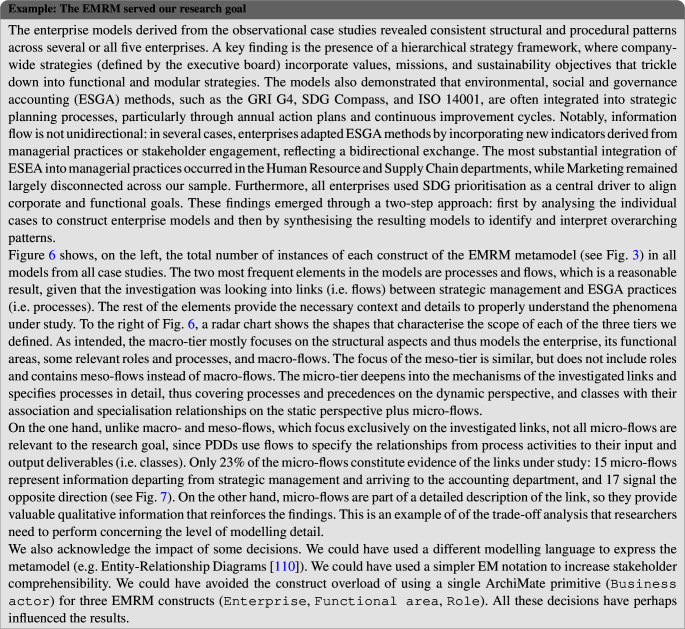

**Fig. 7:**
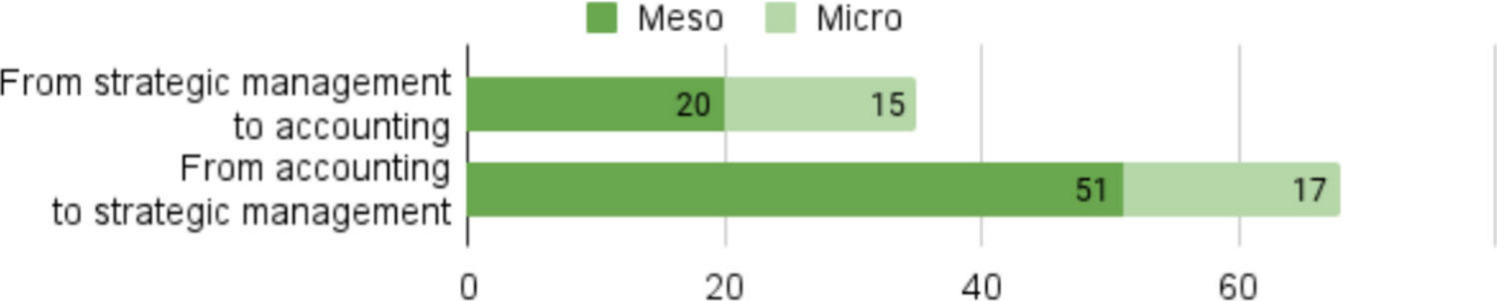
Flows that contribute evidence of the links between ESGA and strategic management practices

7. Document the Research Process and Findings. Comprehensive documentation of the research process, instruments, and decision rationale is critical to ensure transparency, reproducibility, and the scholarly rigour of research project in general [111], and EM studies among them. Researchers should clearly explain how EM was applied at each stage of the methodology; that is, how it structured data collection, facilitated representation of complex organisational phenomena, and supported analysis and interpretation. Explicitly describing the role of EM helps readers understand the logical flow of the research and the rationale behind methodological choices, which enhances the trustworthiness of the study [112].

We recommend emphasising the connection between the models and the research questions, since this strengthens the coherence of the research narrative. This linkage will contribute to demonstrate how the enterprise models serve not merely as descriptive artefacts but as analytical tools integral to answering the research questions and generating insights. We also suggest reflecting on the unique contributions of EM, such as its capacity to integrate multiple perspectives [17] and reveal systemic interactions [113].

Good documentation includes not only detailed methodological descriptions but also reflections on challenges, limitations, and lessons learned throughout the research process [108]. Such reflections support methodological learning within the field and help other researchers anticipate and avoid common pitfalls. Finally, within specific publications, thorough documentation contributes to consolidating a cumulative knowledge base by making research artefacts, including models, accessible and interpretable for future studies, thereby advancing both theory and practice.
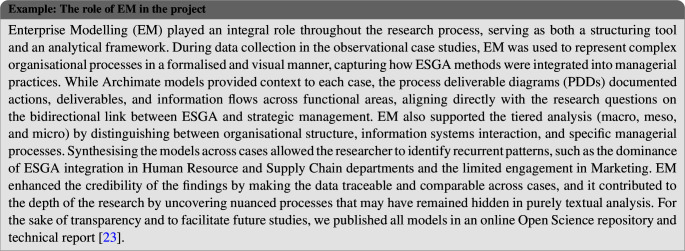


This structured approach should enable EM to be used not only within design or engineering projects, but as a rigorous method for scientific inquiry.

## The opinion of experts in enterprise modelling

Herein, we elaborate on the comments of the experts, grouping them by common themes. We add references when we find that these clarify the argumentation. We identify each interviewed expert with an acronym of the form I#.

### On the applicability of enterprise modelling as a research method

All experts have agreed that EM can be used as a research method. Their reaction ranged from plain agreement to stating that it is *“a very natural thing”* (I3), *“a rather natural proposition”* (I6) or a *“very good idea”* (I8). One expert stated (I4) that *“of course it is a research method or a method for collecting and documenting enterprise knowledge in any kind of situation;”* after all, that *“is one of the core purposes of EM.”* But the experts made two remarks we fully agree with.

- Firstly, the domain of research needs to be an enterprise (I4): EM would not fit a research project in which the phenomena under investigation is not related to organisational structures, actors, processes, business rules, information and/or technology. In fact, the wide array of constructs that are within the focus of EM is a revolving theme in the expert assessment. We also agree with expert I4 when they pointed out that the research project we use as a running example in Sect. 4 does not represent the breadth of opportunities that EM offers as a research method, since the process perspective predominates, leaving other constructs under-represented or directly absent in the metamodel in Fig. 3. Expert I8 pointed out that most EM methods include method fragments focused on information and communication technology (e.g. discovering and modelling the technological components in an enterprise, or aligning the business strategy with the technological strategy). When information and communication technology is not within the scope of the research project, those method fragments are not applicable. We consider that this should not be a problem, as long as the method offers fragments and modelling constructs that suit the needs of the research questions.
- Secondly, to be used as a research method, EM needs to be used to guide the research process, not just to engineer something (I6). This is in line with the proposition of this paper. EM is so often used with the purpose of organisational (re)design and information systems engineering, that its capability to be used as a research method is often overlooked and not sufficiently acknowledged (see Sect. 3.1). In this paper, we are interested in applying EM methods to answer knowledge questions.

One of the experts (I6) wondered for what fields and disciplines would EM be suitable as a research method. for instance, he suggested that EM would be more applicable in Social Sciences than in Natural Sciences. While this sounds like a natural proposition, it remains an open question that is out of the scope of this paper.

One expert (I6) identified similarities and differences between Grounded Theory and Thematic Analysis (on the one hand) and EM (on the other), which we now elaborate further. Grounded Theory and Thematic Analysis are qualitative research methods aimed at systematically analysing data to develop theories or to identify patterns or themes. EM also analyses the information provided by the organisational stakeholders or the company documentation. Often, Grounded Theory and Thematic Analysis involves open coding; that is, initially coding data without preconceived categories. In EM, the coding is implicit, the codes are predefined and they correspond to the modelling constructs. In Grounded Theory and Thematic Analysis, the data and the results typically remain textual, whereas in EM, the results soon become graphical.

A couple of experts also drew parallels with the use of Conceptual Modelling as a research method. For I6, Conceptual Modelling is often part of the research method when data analysis is required; for instance, Conceptual Modelling informs data scientists when developing machine learning systems [114]. It can also be used to synthesise theories [115] or develop taxonomies [116]. For I5, when researchers develop a new theory, it is useful to use Information Modelling or Ontology Engineering to capture the structure of the phenomena under discussion. For this expert, EM offers a more refined modelling approach, in the sense that it offers more specialised constructs (e.g. actors, processes, goals). Under some circumstances, *“it makes sense to use these more refined metamodels [...], more refined ontological commitments to look at the world.”*

Expert I5 also introduced an important element in the discussion. In EM methods, we can distinguish (at least) a way of modelling and a way of working [117]. The **way of modelling** refers to the modelling languages offered by the EM method, and the constructs that are part of those languages. The **way of working** refers to the process of the method; that is, the structure of activities that provides guidance to apply the method. The way of working is typically situation, context, and issue dependent [118]. The expert considered that *“both have roles to play in their own accord,”* and yet sometimes we will need to distinguish them to clarify the discourse. For instance, when a research project needs to understand *“certain processes and how things run,”* then *“it makes sense [...] to do a process model.”* Thus, we could adopt the way of modelling of an EM method that offers the process modelling constructs that are needed to answer the research question. However, the way of working of the selected method might include some practices that are useful during a research project and other practices that are not deemed valid in a research context. For instance, the approaches to elicit information from stakeholders (e.g. interview, observation, participatory modeling workshop), organisational procedures, manuals and policies (e.g. document analysis) might be useful; see [7] for details. But applying some guidelines related to managing and negotiating in situations where conflicts of interest among organisational stakeholders, power relations, and mandates from managers affect the modelling process might be undesirable in a research project. *“In Science, we normally would have the luxury of trying to be more objective. So there might be a stakeholder game, but that’s not desirable in the scientific context.”*

Despite these remarks, the general reaction of the experts to our proposal, first presented in PoEM 2023 [6], is nicely summarised by I5: *“So the [PoEM 2023] paper did not come as a surprise, but it’s good for us to reflect on this [the possibility to apply EM as a research method] and make this a stronger point.”*

We asked the experts to look back at their own past research projects, and reflect on whether they had ever used EM as a research method, consciously or not. Six out of eight experts recalled having used EM in such a way. Some examples were:

- Projects in which only one perspective of EM was applied with a research purpose; e.g. Information Modelling (this was mentioned by I5 and I8).
- During qualitative analyses, by using the constructs of an EM method to code data or transcripts (I2).
- Using EM to create reference models and reference architectures, since these are, in a way, theories. *“A reference architecture has a claim that it works in certain situations”* (I5).
- Using some EM perspectives to create models that are later used to drive the rest of the research; e.g. deriving research questions from the goals present in the enterprise model (I6).
- Creating an EM and then analysing its content to answer the research questions. This is, in essence, similar to the use we made of EM in the project described in Sect. 4. However, the domain described by expert I7 was different. In an applied project, they created an enterprise model that identified organisations, actors, roles, goals and data entities. Then, they run several data coverage queries related to their research questions, such as *“How many providers do we have for this data source? This decision depends on what data sources?”*

Interestingly, all experts had to reflect for some time before answering. After all, they were not necessarily aware of the fact that they were applying EM as a research method. *“When we did that, we did not perceive it as a research method. But, from your perspective, when I look back at what we did, we used enterprise models not so much for enterprise engineering per se, and not so much they were used for information systems design afterwards. But [...] we explored [...] specific research questions”* (I7).

### On the strengths and benefits of enterprise modelling as a research method

We elaborate on the themes emerged from the analysis of expert responses, indicating how many experts have discussed each theme (while this number does not allow to infer any further claims about the theme, we find it informative as an indication of theme coverage).

**EM provides structure to the research process** (N=3). Experts consider that different EM perspectives can help researchers steer a research project in different ways. Firstly, the motivational perspective can aid in defining the research objectives (using goals). Expert I6 envisioned how it would help managing expectations in research projects with many different stakeholders: *“In research projects with multiple stakeholders, where you would need to balance maybe different kind of goals and problems. And where the problem is kind of a messy one. [For instance], that you have a lot of them [goals] and then you need to figure out on what to focus, and what is really the problem and what is a cause. And I think [that], for these kind of early analysis, it would be rather useful.”* Expert I8 not only agreed that EM could help *“to posit [formulate] the research objectives more clearly,”* but also *“to understand better the traceability between research objectives and other aspects”* of the research project. Secondly, expert I6 suggested that the process perspective could be useful to specify the research protocols. Similarly, expert I1 mentioned that, in situations where several several stakeholders need to coordinate efforts during the project, researchers could combine the process, actors and information perspectives to specify which participants (roles or actors) take part and when. Thirdly, for I6, the information perspective (concepts) is useful to inform data analysis research activities, as discussed in Sect. 5.1.

**EM helps eliciting information from sources** (N=4). The way of working of EM, along with its way of modelling, can inform the researchers how to analyse an organisational domain (I5). The EM community has provided guidelines on how to perform document analysis, observation (i.e. ethnographic research), interviews, focus groups, and participatory modelling workshops with organisational stakeholders [97, 119–122]. Some EM methods have some of these guidelines built in their way of working (e.g. 4EM [7]). For expert I2, using such a well-engineered and fold out approach *“helps focus on certain areas and it helps elicit information.”* As expressed by I7, EM *“provides certain mapping of your problem domain and afterwards you can do some sort of analysis.”* The participation of organisational stakeholders becomes a key factor, because it helps uncover special interests that the organisation has and that, otherwise, remain unnoticed. Finally, I3 posed the idea that this strength of EM could be useful beyond the traditional conception of enterprises; it could be applied, for instance, to investigate how medical practitioners are treating patients with a certain illness.

**EM provides structure to the elicited phenomena** (N=6). The primary sources from which organisational phenomena is elicited, typically provide the information in an unstructured way. Enterprise documentation and recountings by organisational stakeholders are typically incomplete, contain redundancy, are not exhaustive in relating elements to each other, contain contradictions, etc. The abstract syntax of EM languages, whether expressed as a metamodel (e.g. the case of MEMO [123]) or a textual grammar (e.g. DEMO [124]), provides a structure of interrelated constructs. Moreover, EM offers an ontological commitment that is useful to elicit and document phenomena about organisations: the way of modelling helps *“to understand the structure of the domains that we want to do [investigate], the things you want to study”* (I5). As a result, creating an enterprise model implies arranging the information elicited from the sources in accordance with such structure. This has a number of benefits, which are reinforced by the fact that the structured information is presented graphically. For instance, it allows to grasp how different elements of the organisational phenomena relate to each other (I4), detect and resolve conflicts or contradictions (I4), detect missing information (I4, I8), detect errors (I8), and establish and verify traceability (I1, I8). This structuring capability is, according to I4, *“one of the largest, biggest advantages of Enterprise Modelling.”*

**EM presents the information graphically** (N=1). Expert I4 associates the benefits of information structuring to the visual presentation of organisational phenomena in the enterprise models: *“I would say that the most important strength of Enterprise Modelling is that you structure your data in a visible way.”* When, during a project, the researcher elicits information from organisational stakeholders via interviews or focus groups, they potentially gather much valuable data. But the format of interview transcripts is natural language (i.e. unstructured text). It can be subject to coding to find patterns or themes. *“But if you take that data and put it in another form which is more summarised and structured, then there’s a visible element and you can see physically how different things relate to each other. I think that is the biggest advantage of Enterprise Modelling in the context that you’re trying to use it”* (I6). The expert illustrated their point based on some experience during a project commissioned by a large company that was using textual documents to specify service requirements and later experiencing problems during service development. The expert then sampled a couple of requirements specifications, and inspected them to identify the actors that will be using the system, the relations among them, the goals that the company intends to achieve with the service, the process underlying the service, etc. Creating the corresponding enterprise models revealed incompleteness and contradictions that had remained unnoticed to that date. *“So I think visualising a lot of information in a smaller space, I think that’s one of the biggest advantages. Which is why it could be used as a data collection and data analysis method”* (I4).

**EM facilitates verifying and validating the elicited information** (N=3). Models can be subjected to verification and validation [125]. According to Chapurlat and Braesch [126], verification and validation naturally follow any enterprise modelling activity in industry settings; we claim that the same should be the case in a research setting. They provide the following definitions. *Verification* refers to detecting and solving (i) inconsistencies of an enterprise model with its EM language (e.g. its metamodel) and (ii) incoherence between two enterprise models which must, for example, respect the same set of properties. *Validation* refers to assuring that an enterprise model provides an accurate and relevant representation of reality and that it also takes into account the requirements coming from organisational stakeholders. Through verification and validation, it is possible to increase the likelihood that the elicited information (i.e. the collected research data) corresponds to actual structure and behaviour of the enterprise. In essence, this is done by having expert modellers and stakeholders review, comment and (in the participatory approach) edit the models until they agree that the model is fit for its purpose and has sufficient quality (e.g. completeness, validity, comprehensibility). For more background on (enterprise) model quality, refer to [7, 125, 127–132]. Expert I4 recalled a project in which an enterprise using chevron diagrams to represent processes. The expert translated these diagrams to process models specified with an EM language and then complemented these with the information perspective (i.e. what information entities are input or output for each activity). Then it was possible to verify the processes to diagnose why some of them were causing problems. According to I8, since enterprise models represent the information explicitly, they can be analysed with different purposes (e.g. detecting errors, ensuring completeness, checking traceability, analysing the alignment between business and information and communication technology). Moreover, the holistic approach of EM (the fact that enterprise models offer many interrelated perspectives of the enterprise) facilitates that organisational stakeholders, after given the chance to comment on and validate the models, end up endorsing them. This is consistent withe the experience of I7, who expressed it as the stakeholders *“buying”* and *“committing to”* the models.

**EM is a validated approach** (N=1). The fact that there are plenty of empirical validations of EM should favour its acceptance. *“Most of it [Enterprise Modelling] is theoretically grounded. And proven to be useful in practice”* (I6). Many publications support this claim; for instance, [58, 133–139]. Of course, the EM community should now demonstrate that EM is valid for research purposes. This paper is a starting point.

**There are plenty of available learning materials** (N=1). As I3 pointed out, not only there are scientific papers on EM, but also books that comprehensively cover the principles, guidelines and languages (e.g. [7, 122, 140, 141]). There are also online tutorials with learning material (e.g. slides, videos and exercises).^4^

### On the weaknesses and challenges of enterprise modelling as a research method

We have distinguished two scenarios in Sect. 3.1, depending on whether the scientific researchers applying EM as a research method belong to disciplines within Information and Computer Sciences or not. Some challenges are common to both situations, whereas others have different relevance in each.

**Enterprise Modelling needs to be learned** (N=5). In general, all modelling languages need some training in order to understand or create models represented with the language. Yet, there are characteristics of the languages or their users that might affect performance in tasks related to models (e.g. see [142]) . Examples of the former are semiotic clarity, perceptual discriminability, semantic transparency and graphic economy, since characteristics pertain to the graphical notations and are expected to increase their cognitive effectiveness (e.g. see  [143]). Examples of the latter are familiarity with the domain being modelled, and their training and education (e.g. see [144]). Potentially, two stakeholder groups need to learn EM during a research project, namely researchers and organisational representatives. We now elaborate on each of them, building upon the insights offered by four of the experts.

**The researchers** act as enterprise modellers and need to be capable of eliciting information from the organisational representatives (and other sources such as enterprise documentation). The researchers also create enterprise models themselves or facilitate participatory modelling sessions and analyse the resulting models to answer research questions. The challenge to learn EM methods seems more pertinent in the case of researchers outside Information and Computer Sciences. Researchers within Information and Computer Sciences will likely have experience in some modelling languages. Research has shown that users of a modelling language of a given EM perspective (say, process modelling) understand equally well models created with another modelling language of the same perspective, even when they have never been exposed to this modelling language before [145]. Researchers outside Information and Computer Sciences might not have experience with graphical conceptual modelling methods. Therefore, learning sufficiently about the EM method to apply it within their research might require a considerable time investment and cognitive effort. As some experts stated, *“if you’re not used to modelling, it is complex”* (I2), *“if you need to model things, you also need to have the expertise in order to be able to model them properly”* (I8); it is not just a matter of dragging and dropping model elements on an empty canvas. With untrained enterprise modellers, the expert envisions potential problems such as using the modelling constructs incorrectly or leniently. It could be the case that some researchers might not have time or interest to familiarise themselves with it EM (I2). Some researchers might only become interested once they have been convinced of the benefits EM brings as a research method (I8). Some experts share the impression that the EM community could take ownership of this challenge and act to facilitate the adoption of EM methods by researchers within or (especially) outside Information and Computer Sciences. They used using expressions such *“this could be a barrier that we need to to tackle”* (I1). Some alternative courses of action will be discussed in for this will be discussed in Sect. 5.4 .

**Organisational representatives** need to be capable of co-creating enterprise models (in the case of participatory modelling sessions), as well as understanding and judging the validity of the resulting models. *“Sometimes, the models are too abstract for some people [...] The models are not accessible to everyone”* (I3). However, many experts have experienced that organisational stakeholders do not need to be trained before participating in EM projects, especially when the modelling language notation is simple and the enterprise modeller has good facilitation skills. *“The more semantics there is in the notation [...] the more difficult it is [to understand the model]”* (I4). Instead, the expert advocates for extremely simple modelling languages. That allows organisational representatives to focus on discussing about their organisation, rather than deciphering models.

**The proper Enterprise Modelling method and constructs need to be selected to fit the research project and questions** (N=7). There needs to be a good fit between the Enterprise Modelling method and the research project or discipline. Since there are plenty of EM methods, a selection process is needed (I6). *“I also think that the first question to ask oneself is which types of questions do I need to answer? Is it a ’what’ question? Is it a ’how’ question? Is it a ’why’ question? Is it a ’who’ question? And then choose a method from there, not being strictly confined to one specific method. Because I think that’s counterproductive”* (I4). It might be the case that the selected EM method needs to be *“tailored to the research purpose”* (I2). EM languages tend to have a significant amount of modelling constructs. Several researchers have expressed the concern that, given a research project, only a subset is needed; that is, there needs to be a good match between the organisational concepts that the research project requires investigating and the selected modelling constructs (I1, I2, I3, I4, I5). For instance, the researcher needs to decide about concepts (e.g. whether they need to model actors, goals, processes, rules, information, technology) and about relationships (e.g. whether it is relevant to relate processes and goals). One expert has suggested that the research goals and questions are key to make this decision (I8). Expressing this challenge in terms of ontological method analysis, every research domain has an implied ontology that needs to be made explicit, that is comprised by a set of ontological constructs [146]. The EM language offers modelling (or grammatical) constructs to express the domain, and the researcher needs to select a subset so that, (i) for each ontological construct, there is at least one modelling construct and, (ii) there is no modelling construct that does not correspond with any ontological construct. If *i* is not satisfied, the researcher faces a construct deficit (part of the research domain cannot be modelled, compromising the researcher capability to answer a research question completely), whereas if *ii* is not satisfied, they face a construct excess (potentially including elements in the enterprise models that are not necessary to answer the research questions, also making them more complicated for stakeholders to understand). In an ideal situation, the mapping between ontological and modelling constructs is one-to-one, or otherwise the models might be more difficult to understand or interpret, since they might face construct redundancy (when two modelling constructs can be used to model a single ontological construct, potentially leading to inconsistencies in the way one type of domain element is represented in the models) or construct overload (when the same modelling construct is used to represent different ontological constructs, potentially causing ambiguity and therefore difficulty in interpreting the model accurately). The tailoring of the EM method to the research project needs to be supported (I8), especially for researchers with little EM experience.

**The modelling constructs can bias the investigation and interpretation of the phenomena** (N=3). The selection of modelling constructs discussed above constitutes the ontological commitment [78]. Informally, it constitutes the lens with which the organisation is inspected. As with any lens, it becomes suitable for some purposes but it also introduces some distortion. *“So for the way of modelling, the biggest risk I see is ’the hammer and nail’ aspects, in terms of the ontological commitments. That it [could] just enforce a certain way of thinking and put people in the wrong perspective”* (I5). Other experts have expressed similar concerns; researchers could happily resort to the available modelling constructs to structure the observed phenomena without critical thinking, or even overlook important phenomena when the EM method does not highlight them in its language (I2). This issue already happens when EM is applies in consultancy, but if we *“apply it in Science, we have to take even more responsibility of what we are doing:”* that is, researchers need to be aware of and transparent about the ontological commitment of the EM methods they use and its effects on their research (I5). Nonetheless, as expert I6 commented, such type of bias is not exclusive of modelling methods, but can also occur while applying other research methods. For instance, when conducting Thematic Analysis, there is a risk of harbouring assumptions about the themes present in the data. And when conducting Grounded Theory, a bias can occur when the researchers have preconceptions about the codes to use.

**Enterprise Modelling requires a maturity process to be used effectively.** (N=5). Several experts caution against underestimating the dexterity required to apply EM properly, especially as a research method. They have warned of several pitfalls that would hinder the ability to answer the research questions accurately. For instance, paying attention to the modelling notation but not to the way of working (I4), selecting the wrong organisational stakeholders to elicit information from (I4), or focusing on modelling constructs meant to represent entities of the organisational domain while neglecting the modelling constructs meant to represent relationships among the former (I2). Researchers could apply EM inappropriately or shallowly. For instance, some formats of participatory EM sessions involve sticking small pieces of paper to a plastic sheet in the wall; *“often people think [that] any time you put something on the plastic sheet [this] is going to be Enterprise Modelling”* (I6). However, the expert extended this risk to virtually any research method, likening it to situations where novice researchers do not apply Thematic Analysis rigorously: *“they put some tables and they put some things in the tables and say ’Look, there’s themes and there are the codes of some themes,’ without having deeper thinking”* (I6). To avoid such pitfalls, expert I4 envisioned that researchers applying EM as a research method would first discover the capability of structuring and representing information about the organisation with the modelling language notation, then they would try to improve the appearance of the models, afterwards aiming at how faithfully represents the organisational phenomena, and finally reflecting on how well the model achieves its purposes. *“And that is a maturity ladder that comes with time and experience. And the the biggest trap is to think that notation, the modelling language, will solve all your problems. [...] It won’t. So failing to understand that Enterprise Modelling is much more than just a language, a notation, that is the real trap in this”* (I4).

The conception that organisational representatives have of EM is also susceptible to an improvement process. Enterprise modelling requires much commitment from them; they need to provide plenty of information to the enterprise modellers, sometimes engage in collaborative modelling, validate the resulting models, reflect on their implications and consequences. Often this requires several sessions. And this, according to expert I7, is problematic, since they might not be willing to invest the necessary resources and time. And the expert considers that this challenge might affect researchers from disciplines outside Information and Computer Science more profoundly.

On the one hand, as we have argued above, enterprise models suffering from incompleteness or containing invalid elements can hinder their utility for answering knowledge research questions. On the other hand, there is a risk in modelling more aspects of the domain than are needed to answer the research question, hindering the efficiency and utilising more resources and time that would be strictly necessary. Expert I8 calls for finding the right balance. The level of detail in the enterprise models needs to be in a trade-off with situational factors of the research project, the expected research outcome, the available time, etc.

**The subjective nature of the information being elicited is a challenge to scientific research** ($\hbox {N}=4$).

It is reasonable to assume that researchers will want to apply EM within both positivistic and interpretative research traditions.^5^ From a *positivistic* or *realist* perspective, EM serves as a means to construct models that represent reality, which can subsequently be tested and refined through empirical confrontation. This approach assumes the existence of an objective reality that the modelling process seeks to capture. By contrast, an *interpretative* or *constructivist* stance emphasises the plurality of stakeholder perspectives and the ontological assumptions they bring to the modelling process. As with other research methods such as case study, both approaches are possible, but they differ significantly in their assumptions, goals, and procedures.

However, expert I4 warns against considering that enterprise models bear the truth about the organisational domain under investigation: *“all models are dependent on when they’re done, who is doing them, and who is giving input to the models.”* For this expert, epistemologically, EM requires constructivism rather than realism. Experts also argue that EM often entails negotiating with and achieving sufficient agreement among organisational representatives observing the enterprise from different perspectives, occupying positions in different hierarchical levels, belonging to different departments, or having different agendas (I5): *“whereas, in Science, we normally would have the luxury of trying to be more objective. So there might be a stakeholder game, but that’s not desirable in the scientific context. [...] We cannot just blindly apply our ways of working in a scientific context. Because there really is a fundamental shift away from negotiation and intersubjectivity and towards the quest for objectivity.”* In the event that two or more actors in a project insist on divergent conceptions of the organisation, the modeller faces the challenge of whether to prioritise certain perspectives, merge them, or acknowledge irreconcilable differences. Such decisions may align with, or contradict, the empirical data collected, and might facilitate or complicate answering the research questions rigorously.

Expert I7 agrees that enterprise models carry subjectivity, and I5 misses *“guidelines built in the methods to find this objectivity,”* and calls for extending EM methods with *“warning systems, to say ‘Here you run the risk of being subjective’.”* The inherent subjectivity in models hampers the replicability of the research results (I3). In principle, this challenge can be alleviated by applying triangulation; that is, having several pieces of evidence supporting the same conclusion. However, a thorough triangulation or validation of an enterprise model is often too time-consuming (I7).

### Promoting the recognition of enterprise modelling as a research method

There seems to be a general agreement that establishing a new research method takes time; scientists are rather conservative when it comes to accepting such novelties (I5), and marketing a new research method is a challenge (I6). Experts I2 and I4 recalled that the acceptance of Design Science as a research methodology took some time to gain widespread recognition and adoption within the academic community. But that it can be inspirational to look into the process through which other research methods became accepted. Apart from Design Science [26], I2 mentioned Action Research [148], Case Study research [149], Qualitative Data Analysis [150]. And I1 mentioned Systematic Literature Reviews [151] and Mapping Studies [152]. The experts recommended a step-wise approach, with I5 outlining two coarse-grained phases intended to build a track record: (1) collaborate with researchers (especially outside Information and Computer Sciences) to demonstrate the performance of EM as a research method, and (2) report on the experiences and the advantages brought by EM (e.g. data or insights not revealed by other research methods under similar circumstances). Herein, we elaborate on concrete actions that the EM community can pursue.

**Adapt Enterprise Modelling to be better suited for research and align it with other research paradigms or methods** (N=6). Some discussions reported above underscore the need to adapt EM to act as a research method. For instance, preventing the use of (or providing proper methodological guidance in) techniques that bear risks in research contexts, such as stakeholder negotiation upon competing viewpoints on the organisation. Or extending EM methods with mechanisms to detect subjectivity. Additionally, I2 has suggested adapting and proposing EM as a variant for conducting qualitative analysis. For instance, EM modelling constructs would predefine a set of codes with which elicited data could be structured and analysed, as we have done in the project reported in Sect. 4. It could also be convenient to adapt EM to some research methods that are specific of or widely applied in disciplines outside Information and Computer Sciences. But, as expert I3 recommended, the EM community should be proactive, rather than wait for the researchers in those areas to take the first step. Two experts (I6 and I7) have recommended to conduct a comparative study to align EM with the activities of Design Science, identifying in which of them EM is a proper way of working. Additionally, expert I7 has recommended aligning EM with methods from business-oriented disciplines (e.g. Business Model Canvas [35]). Finally, expert I5 sees opportunities in the crossovers between Enterprise Modelling and other research methods. For instance, Group Model Building (with which EM shares the challenge to manage subjectivity) and System Dynamics (where Stock and Flow Diagrams could complement EM in addressing complex societal and environmental challenges).

**Facilitate the adoption of Enterprise Modelling as a research method by providing learning and support materials** (N=3). Nowadays, the Internet provides many formats to design and deliver learning material that is affordable and engaging. The EM community could collaborate on creating such knowledge base, paying attention to its visibility and to lowering the learning curve for researchers outside Information and Computer Sciences (I1, I3). We can distinguish three categories. The first one is intended to emphasise the benefits of EM as a research method and motivate researchers to learn more; for instance, leaflets, posters, short videos and case study reports. The second category aims at training the researchers to their desired capability level; for instance, online tutorials, instructional videos, course books, and massive online open courses. Finally, one category is meant to support researchers while applying EM in research projects; for instance, cheat-sheets, handbooks, templates, toolkits, and community forums. Naturally, the three categories overlap. And they could also be intended for practitioners.

Expert I4 proposed using simple modelling languages and notations that reduce the need training, so the focus can be put instead on the way of working. “Because the more semantics there is in the notation, particularly linking concepts to each other (e.g. if there is a double arrow that means this , or a star [*] means something)... The more things like that, the more difficult it is [to understand the model].” I4 suggests using intuitive labels and textual notes to express such semantics. Only after a model has been validated by the organisational representatives, would the model be expressed with a more formal syntactic notation, but also only when this is absolutely necessary; for instance, when the more formal model will be the input to automated processing (e.g. verification or simulation) or to an information system development process.

As recommended by I1, if PhD students are trained in EM during their doctoral education, they will more easily resort to it during research project design. However, this seems easier to do within Information and Computer Science doctoral programmes than outside this field. At least until EM gains more recognition as a research method.

**Write scientific publications about using Enterprise Modelling as a research method** (N=6). To achieve a proper recognition, scientific publications are necessary. Several experts have recommended publishing the results of case studies or technical action-research projects applying EM as a research method (I2, I4, I7, I8).

With respect to the content, we have received suggestions about showcasing how EM can be used alongside Design Science (I7), and about using cases from fields outside Information and Computer Sciences to illustrate the differentiation of EM with respect to their conventional research methods (I4, I8). Expert I5 recommends analysing papers from a community outside our discipline and applying our methods to them. This would (perhaps) prove the added value of EM by showcasing that the results can be better presented as an enterprise model, highlight inconsistencies or incompleteness, or revealing overlooked findings. The expert invited EM researchers *“closer to the end of their career”* to participate in this endeavour, given their experience, time and resources.

With respect to the outlets, there are trade-offs between the likelihood of getting the papers accepted and the visibility they might have. For instance, conferences focused on modelling might be more inclined to publish those papers but they might have less visibility than journals focused on wider disciplines such as information systems or software engineering (I2). Experts I5 and I7 suggest writing a paper in research-method tracks of conferences or any other outlet focused on research methods, especially those outside our discipline. And still any of these approaches might not create awareness in fields outside Information and Computer Sciences. To achieve proper awareness and recognition, two experts have argued that a reference book that presents EM as a research method is needed (I2, I6). Similarly to books about Case Studies, Thematic Analysis, or Statistics, this book should not give the impression that EM is solely for information and computer scientists. Finally, a plateau of recognition is reached when other research methodology books cite EM as a research method or also elaborate on this.

**Collaborate in research projects outside Information and Computer Sciences** (N=3).

Several experts highlighted the importance of engaging with research communities beyond Information and Computer Sciences to demonstrate the value of Enterprise Modelling (EM) as a research method. Expert I8 suggested that collaboration should not be limited to theoretical advocacy but should involve concrete application of EM in research contexts that are traditionally outside our discipline: *“So you have a researcher from another domain that collaborates with you and applies this [Enterprise Modelling] as a research method. A researcher or someone from industry”* (I8).

However, experts caution against expecting external researchers to adopt EM independently by relying solely on written materials. As expert I4 remarked, *“you can’t learn Enterprise Modelling from a book [...] only. So I would say [that we should] join their projects.”* The expert envisions a facilitator role for EM specialists within interdisciplinary research teams, where researchers outside our field formulate their research question within a given research project (including its partners, cases, etc.). And EM experts help them conduct the Enterprise Modelling parts of their project. *“To make them see for themselves within their own projects what the benefits [of EM] are.”*. This facilitator approach emphasises experiential learning and hands-on collaboration as the primary means of proving EM’s added value.

Another proposed strategy involves incremental integration of EM capabilities into existing research practices in other domains, particularly those already using modelling-based approaches. Expert I5 described an approach starting with concepts from ontology engineering and information modelling, gradually introducing richer perspectives from EM: *“If you take a project or research effort that is clearly out of our scope, and you use some of those techniques to see if we can provide more insight, create more awareness... As a strategy, from there we can grow and start introducing the more refined metamodels.”* For instance, in fields employing System Dynamics, the expert sees opportunities to complement existing models with actor-oriented or process-oriented views, thereby broadening the analytical scope.

Finally, one expert proposed a pragmatic route for increasing EM’s visibility within other scientific disciplines. I5 suggested becoming involved in a project outside the Information and Computer Sciences field, and letting another researcher apply their traditional approaches. Then *“we can say ’Wait a minute! We have techniques that provide added value, and [that are] also defendable from a scientific insight point of view.’ And then we can start working with those researchers to infiltrate their papers with our kinds of [EM] approaches.”* (I5). This pathway entails demonstrating EM’s utility in established research problems, fostering gradual adoption through collaborative publications.

**Disseminate and discuss the idea in scientific events and social networks** (N=2).

Experts I3 and I5 made some recommendations for disseminating the applicability of EM as a research method. For instance, (i) organising tracks or workshops focused on this, within EM or Conceptual Modelling conferences, (ii) presenting cases in conferences and workshops of other fields (especially in research method tracks of conferences outside EM), (iii) promoting the idea in social networks, (iv) delivering a webinar, and (v) establishing a grant or contest to stimulate or award the application of EM as a research method (especially outside Information and Computer Sciences).

### Overcoming reviewer and reader resistance in the short term

Before EM gains recognition as a research method, we anticipate certain resistance from reviewers and readers of papers reporting on research projects in which apply it (see Sect. 3.1). While discussing this concern with the experts, they agree that the risk of getting a paper rejected for this reason is high, especially in fields outside Information and Computer Sciences, and they have provided tips to minimise the chances of this happening.

In principle, papers reporting on the results of unconventional research methods rather include the rationale for this decision and properly describe the protocol. However, if explaining Enterprise Modelling in the research section of a paper might be difficult (I1), this would become even more challenging for an audience outside our field (I8). The advice was forceful: don’t call it Enterprise Modelling, just do it! (I2, I4, I5). One approach is to combine it with existing research methods. EM can be presented as an aid for Qualitative Data Analysis (I2, I6); for instance, as a way of coding in search of themes or patterns (see a discussion in Sect. 5.1 and how this was done in [3]). EM could also be presented as a data collection technique; for instance, as a way of eliciting information about organisational phenomena more effectively during interviews or case studies. The arguments to incorporate EM during data collection could be linked to the nature of the information that the research questions require eliciting and analysing (I4); for instance, (i) when trying to understand why something takes place in an organisation, then the motivational perspective is relevant (e.g. goals), (ii) if we need to understand what an organisation is dealing with, the information perspective comes into play (e.g. documents, concepts or data entities), and (iii) if we need to discover specific practices or how something is achieved, then the process perspective is convenient (e.g. activities and roles). This becomes the input for selecting the modelling constructs. To avoid overwhelming the reviewers and readers, the notation should be simple and self-explanatory (I4). When interrelating several perspectives is required, the arguments in favour of EM are stronger. Nonetheless, the trick might be to not call EM a research method yet.

These approaches introduce EM surreptitiously: *“And then it becomes, in a positive sense, a Trojan horse”* (I5). Only after a few papers applying EM as a research method have been published following this approach, within a given discipline, then expert I4 would recommend reflecting openly about the role of EM in such research.

## Discussion of the results

Our application of EM as a research method (the running example in Sect. 4) was published in [6] before the expert assessment interviews took place (Sect. 5). This has allowed experts to not only reflect upon their own experience with EM methods and with research projects, but also upon our own results of applying EM as a research method.

Earlier research had highlighted the value of sustainability reporting results for strategic management or the effect of strategic management practices in shaping the firm’s sustainability agenda, in the areas of supply chain management, human resource management or marketing (see, for instance, [153–155], respectively). Our results during the exemplar project complement theirs by showing how enterprises perform the processes that link both areas, and depict in models the exact mechanisms that earlier papers discuss broadly or merely hypothesise. Such a contribution was only possible through the use of the EM, the reason why we claim that the EMRM was effective. It allowed us creating detailed models, rich in qualitative information, that allowed to deepen into the investigated phenomena and reveal non-evident links. The main strengths we perceived here (i) the structure that the EM method provided during elicitation and (ii) the expressiveness of the EM languages. This is consistent with the experts opinion, since most experts also highlighted these facts.

The expressiveness of EM methods also proved to be a challenge, and we had to constrain the set of modelling constructs we used. This has also been reflected in the discussions with the experts. They have remarked the importance of adapting the set of selected constructs to the needs of the research project, rather than applying the EM languages in full. And also the need to clearly acknowledge that such set of constructs constitutes the ontological commitment with which the organisational phenomena is investigated. Another weakness we perceived is the difficulty to formulate quantitative hypotheses. In scientific disciplines such as Cleaner Production, Sustainability Reporting, or Strategic Management, reviewers and readers are used to quantitative research (e.g. structural equations or multiple regression models). The effort to obtain sufficient data points through EM would be prohibitive. Even though qualitative studies are sometimes published in those disciplines, it is unlikely that the reviewers and readers have heard of EM. Convincing the audience of the validity of applying EM as a qualitative research method might prove a challenge, with the consequent risk of having papers rejected.

The experts have not thoroughly discussed the extent to which software tools support EM as a research method. Many tools exist (e.g. ARIS, Sparx Systems Enterprise Architect, and iGrafx). They typically offer structured environments for capturing and visualising complex enterprise structures and processes. These tools could be particularly beneficial in research contexts that require formalised representations, enabling consistent documentation and analysis of organisational elements. The analytical capabilities of EM tools, including simulation, process analysis, and scenario testing, could also facilitate the exploration of “what-if” scenarios and the testing of hypotheses regarding organisational processes or systems behaviour. However, limitations exist. For instance, some EM tools can be rigid, favouring predefined templates over exploratory or qualitative research approaches, potentially limiting creativity in model representation or novel conceptualisations. Additionally, high-end tools may require significant training, making them less suitable for exploratory studies or research with participants unfamiliar with EM software. Furthermore, the focus of these tools is often on operational modelling rather than the interpretive or investigative aspects that research may demand, such as capturing tacit knowledge or informal workflows.

The findings and recommendations presented in this paper should be interpreted considering several limitations and potential threats to validity. Following established guidelines [156], we discuss these in terms of construct, internal, external, and conclusion validity.

**Construct Validity** concerns the degree to which the study accurately reflects the concepts it intends to investigate. One potential threat lies in the *inadequate pre-operational explication of constructs*. The notions of “using EM as a research method” and “conditions for its effective application” are complex and multifaceted. Although we derived these constructs from literature and expert experience, their operationalisation through interview questions and coding schemes may not have captured all relevant dimensions. Furthermore, as EM is understood differently across communities, the interpretations provided by experts might not align fully with other conceptions of EM. Another related threat is *mono-method bias*. Our primary empirical basis for assessing the proposal consisted of semi-structured interviews. Although these were complemented by reference to prior literature, the lack of alternative empirical strategies (e.g. observation of research projects applying EM, beyond our exemplar project) may have limited the breadth of evidence.

**Internal Validity** addresses whether the observed insights can be attributed to the proposal itself rather than external factors. The expert assessment was based on interviews, which can be subject to *experimenter expectancy*. Given that the researchers designed both the proposal and the interview guide, there is a risk that unintentional cues or framing influenced participants’ responses. Similarly, *hypothesis guessing* may have occurred, as experts were aware that the purpose of the study was to advocate for EM as a research method, potentially biasing their opinions towards a positive assessment. The study relied on voluntary participation by experts, introducing a potential *selection bias*. Those who agreed to participate may hold more positive attitudes towards EM than the broader community. Also, while their expertise in EM contributes to their credibility, it may also introduce a *confirmation bias*, as experts already familiar with EM could be predisposed to view its adaptation to research contexts favourably.

**External Validity** relates to the generalisability of the findings. The interviews involved a small number of experts (eight), primarily drawn from the EM research community. Their perspectives may not reflect the views of researchers in other domains, who represent a key audience for the proposed approach. Consequently, the results should not be assumed to generalise to all disciplines, organisational contexts, or research settings. Additional empirical studies involving interdisciplinary research teams would be necessary to validate and refine the proposal.

**Conclusion Validity** refers to the degree to which conclusions about relationships are reasonable. As the findings are largely interpretative, based on thematic analysis of expert interviews, there is a risk of *interpretation bias*. Although we applied coding procedures, qualitative analysis inherently involves subjective judgement. Furthermore, the absence of triangulation with other data sources limits the robustness of the conclusions.

Overall, the experts are confident about the applicability of EM as a research method. However, the actual value of EM as a research method will (likely) become evident when more researchers from outsider disciplines apply such an approach, and this has been noted as a key aspect to achieve wider recognition within and especially outside Information and Computer Sciences. The selection of the appropriate EM method for a given project is sensitive to the results of the initial problem investigation, as well as to the modelling skills of the researcher. EM is not part of most curricula in Economics, Industrial Engineering, etc. It might become necessary to provide guidance to researchers who are inexperienced with EM, before they can fully tap into its potential. The experts have provided clear recommendations in this regard.

## Conclusions and future work

In this paper, we champion enterprise modelling (EM) as a valid qualitative research method. And we claim that it is applicable both within and outside Information and Computer Sciences. By referring to EM as a research method, we mean that it can not only be used to engineer enterprises and information systems, but also to collect data, analyse it and respond to research questions. We have proposed when and how to apply an EM method as a research method, illustrating our proposal with a running example. We have consulted experts in EM. All agreed to recognise the applicability of EM as a research method, identified its strengths and weaknesses, and made recommendations to promote the recognition and application of EM as a research method within and outside Information and Computer Sciences. As future work, we aim at applying EM as a research method in more research projects, with different types of research questions. To test means of generalising the results obtained through an EM research method, we would attempt techniques such as the method comparison approach [157] or the inductive reference enterprise architecture modelling [158], which allow producing reference models from case models. Investigating the appropriateness of current EM software tools and how they could be extended to better support using EM research method is worthwhile. Additionally, the expert recommendations delineate a roadmap of future work. We consider that EM constitutes an untapped potential for researchers from a diverse set of fields, who could incorporate it in their research methods toolkit. One of the experts we interviewed expressed: *“I am very excited about thinking of Enterprise Modelling as a scientific method”* (I5). We share this excitement and we hope to soon find that Enterprise Modelling becomes a known and accepted research method. In the meantime, we encourage researchers to share their experiences, and call for the Enterprise Modelling community to jointly promote this idea.

## Footnotes

1 While not a full-fledged EM method, we considered the authors’ research approach representative of the intentions we pursue in championing EM as a research method.

2 Younger researchers came into contact with EM once the discipline was already established, allowing them to estimate their years of experience straightforwardly. More senior researchers witnessed the emergence of EM, a discipline that began taking shape around 35 years ago [60]. It was during this time that they began actively participating in defining EM foundations and methods.

3 Originally intended for information systems development methods, it is currently used for other types of methods and processes (e.g. we have used it to model many ESGA methods [68])

4 For instance, https://merode.econ.kuleuven.be/slides/index.html and http://dx.doi.org/10.13140/RG.2.2.35214.00326

5 The reader can refer to [147] for a comparison of various epistemological frameworks.
